# Renal function in HIV-infected children and adolescents treated with tenofovir disoproxil fumarate and protease inhibitors

**DOI:** 10.1186/1471-2334-12-18

**Published:** 2012-01-23

**Authors:** Giuseppe Pontrelli, Nicola Cotugno, Donato Amodio, Paola Zangari, Hyppolite K Tchidjou, Stefania Baldassari, Paolo Palma, Stefania Bernardi

**Affiliations:** 1 University Department of Pediatrics. Ospedale Pediatrico Bambino Gesù, Piazza Sant'Onofrio, 4, 00165 Rome, Italy

## Abstract

**Background:**

Kidney disease is an important complication in HIV infected people, and this may be related to infection or antiretroviral therapy (ART). Our aim is to assess renal function in HIV infected paediatric patients, who may be particularly affected and are likely to take ART for longer than adults, and investigate the long term role of Tenofovir Disoproxil Fumarate (TDF) alone or co-administered with Ritonavir-boosted Protease Inhibitors (PI).

**Methods:**

Serum creatinine, phosphate and potassium levels, with estimated Glomerular Filtration Rate (eGFR), had been prospectively evaluated for 2 years in a cohort of HIV infected children and adolescents (age 9-18) on ART, and data analyzed according to the exposure to TDF or simultaneous TDF and PI.

**Results:**

Forty-nine patients were studied (57% female, mean age 14). Sixty-three percent were treated with ART containing TDF (Group A), and 37% without TDF (Group B); 47% with concomitant use of TDF and PI (Group C) and 53% without this combination (Group D). The groups didn't differ for age, gender or ethnicity. The median creatinine increased in the entire cohort and in all the groups analyzed; eGFR decreased from 143.6 mL/min/1.73 m^2 ^at baseline to 128.9 after 2 years (*p *= 0.006) in the entire cohort. Three patients presented a mild eGFR reduction, all were on TDF+PI. Phosphatemia decreased significantly in the entire cohort (*p *= 0.0003) and in TDF+PI group (*p *= 0.0128) after 2 years. Five patients (10%) developed hypophosphatemia (Division of Acquired Immune Deficiency AE grade 1 or 2), and four of them were on TDF+PI.

**Conclusions:**

Renal function decrease and hypophosphatemia occur over time in HIV infected children and adolescents on ART. The association with co-administration of TDF and PI appears weak, and further studies are warranted.

## Background

Antiretroviral therapy (ART) has reduced morbidity and mortality in HIV infected children as in adults [1,2], but not much is known on the effect of long-term exposure to ART in children, because registration studies have a limited observation time and are often not focused on adverse events related to drug interactions [3]. Kidney disease is an important complication in HIV infected people, and associated to increased risk of morbidity and mortality, related to viral infection (as in HIV associated nephropathy, HIVAN), or to ART [4].

Tenofovir Disoproxil Fumarate (TDF) is a Nucleotide Reverse Transcriptase Inhibitor, which is considered a component of recommended regimens in major Clinical Guidelines for adults, for its efficacy, activity against hepatitis B, and availability in co-formulation allowing a single pill a day regimen [5]. TDF has a good safety profile but several studies highlighted its association with renal damage in adults, with cases of severe tubular dysfunction characterized by elevated creatinine, hypophosphataemia, hypokalemia, and more rarely Fanconi syndrome which may persist after withdrawal of the drug [6-12].

TDF is not licensed in Europe in patients less than 18 years of age but, by 2010, it received Food and Drug Administration (FDA) approval for use in patients aged 12-18 years and weighing more than 35 kg. Nevertheless TDF has been already used as salvage therapy in paediatric patients for several years, often in association with boosted Protease Inhibitors (PI), and few reports described the use of this drug in children. Two studies determined a favourable safety profile for this drug [13,14] whereas renal toxicity and bone density loss were reported by other studies both in adults and children [15-18].

Renal toxicity seems related to the mechanism of clearance of TDF which involves a glomerular filtration and a tubular phase of active secretion. During the tubular phase, Organic Anion Transporters (OAT1 and OAT3) are involved in the intracellular accessing of TDF, on the other hand, Multidrug Resistance associated Protein (MRP2 and MRP4) regulate the extracellular clearance of the drug [19]. Ritonavir and all PI, could have a significant role as cofactor in the pathogenesis of TDF-induced kidney damage. TDF clearance is slower in subjects receiving PI [20], with an increase of TDF-AUC ranging from 22 to 37% [21] and an accumulation of toxic drug levels in tubular cells [22]. The results of in vitro studies also suggest that protease inhibitors may inhibit the function of the MRP2 and MRP4 membrane transporters, and therefore increase the tubular concentration of TDF, and thus mitochondrial toxicity [23].

Our aim is to evaluate renal function in HIV infected paediatric patients on antiretroviral therapy (ART) and for the first time in a controlled study, the role of TDF and the concomitant use of TDF and PI.

## Methods

A prospective observational study was conducted among a cohort of HIV infected children and adolescents (< 18 years of age) on ART followed between 2008 and 2010 at Bambino Gesù Children Hospital of Rome, Italy. The study was approved by the local Ethical Committee, and informed consent obtained from the parents or guardians of all participants. The aim of the study was to investigate the role of TDF and concomitant use of TDF and PI on renal function in HIV infected children and adolescents. The cohort was therefore analyzed firstly using two groups according to the exposure to TDF (Group A: TDF) or not (Group B: no TDF). Secondly a further analysis was performed according to the exposure to concomitant use of TDF and PI (Group C: TDF+PI) or not (Group D: no TDF+PI). Since TDF was used only in drug experienced children older than nine, we studied only patients above this age, in order to not introduce any age-related bias. Data on gender, age, ethnicity, CD4 T cell count and HIV-RNA (with a value of < 50 copies/mL defined as undetectable) were collected at baseline. Renal function was assessed at baseline, and after 1 and 2 years by evaluation of serum creatinine (assessed with non-enzymatic CREA2 method, a modified version of Jaffé reaction), phosphate, and potassium, and filtrate was estimated by eGFR (estimated Glomerular Filtration Rate) calculated with the Schwartz formula (k*height (cm)/creatinine (mg/dl); with k = 0.55, or 0.70 in males over the age of 13 years). No change in the analytical methods to measure serum creatinine or other markers took place during the study period. Reduction of renal function was classified according to the guidelines of the National Kidney Foundation: "mild" 60-89; "moderate" 30-59; "severe" 15-29; "renal failure" < 15 ml/min per 1.73 m^2^. Hypophosphatemia was divided into four grades according to Division of Acquired Immune Deficiency (DAIDS) scale. For patients under 14 years old: "mild": 3.0-3.5; "moderate" 2.5-2.9; "severe" 1.5-2.4; "potentially life-threatening" < 1.5 mg/dL. For patients older than 14: "mild": 2.5-3.0; "moderate" 2.0-2.4; "severe" 1-1.9; "potentially life-threatening" less than 1.0 mg/dL.

Data for continuous variables were expressed as mean and standard deviation (SD) if normally distributed, or median and Inter-Quartile Range (IQR) if not normally distributed. Categorical data were showed as counts and percentages. Analysis was conducted using Epi Info version 3.5.1 (Centers for Diseases Control, Atlanta) or GraphPad Prism version 5 for Windows (GraphPad Software, San Diego California USA). Statistical analysis of baseline variables was carried out for continuous data with either ANOVA method for Analysis of Variance for normal distributed data, or Mann-Whitney test for non-normal distributed data, whereas chi-squared or Fisher exact tests were used for categorical data. Analyses of variables of renal function were performed with non-parametric Friedman test for comparison of values of baseline, first and second year in each group, with Wilcoxon matched-paired signed rank test for comparison of two time points, and with Mann-Whitney for analysis of values of Group A versus Group B, and values of Group C versus Group D at each time point. A two tailed P value of less than 0.05 was considered significant in all the analysis.

We expected an enrollment of about fifty patients, and about two-thirds exposed to TDF, and half to TDF + PI. Therefore, for eGFR analysis, the power to detect a difference of 15 mL/min/1.73 m2, considering a 95% Confidence Interval, and assuming a Standard Deviation of 20, was 96% in the whole cohort, 86% in Group A, 59% in the group B; 76% in the groups C and D. The power to detect a difference of 15 mL/min/1.73 m2 between Group A and B is 71%, and 76% between Group C and D. Regarding phosphatemia, the power to detect a difference of 0.5 mg/dL considering a 95% Confidence Interval, and assuming a Standard Deviation of 0.7, was 95% in the whole cohort, 83% in the Group A, 55% in the group B; 71% in groups C and D. The power to detect a difference of 0.5 mg/dL between Group A and B was 67%, and 71% between Group C and D.

## Results

Forty-nine patients (9-18 year old) were studied. They had a mean age of 13.6 years, and 28 (57%) were female. The mean CD4% value was 27.1 (± 10.2), corresponding to value of 565 cells/μL; 47% had HIV-RNA undetectable, and 39% of patients had a history of CDC (Center for Disease Control) class C clinical diagnosis, and no patients had creatinine > 1 mg/dL at baseline. None of the patients was treatment naive.

Sixty-three percent were treated with antiretroviral (ARV) regimens containing Tenofovir (Group A: TDF), and 37% without TDF (Group B: No TDF).

Forty-seven percent were treated with regimens containing both TDF and PI (Group C: TDF+PI), and 53% without this combination (Group D: No TDF+PI). Specifically, 16% of patients were on TDF without PI, 20% were with PI but alternative NRTIs, and 16% with other regimens containing neither TDF nor PI. The children didn't differ for age, gender, frequency of undetectable HIV-RNA, or ethnicity (*p *> 0.05 for all comparisons) both between Group A and Group B, and between Group C and D. CD4 resulted lower in group A and Group C if compared to Group B and D respectively (Table 1).

**Table 1:** Baseline characteristics of the patients, overall and in each treatment group

	Total n.49	Exposure to TDF			Exposure to concomitant use of TDF and PI		
		
		A TDF n.31 (63%)	B NO TDF n.18 (37%)	p	C TDF+PI n.23 (47%)	D NO TDF+PI n.26 (53%)	p
**Female **n (%)	28 (57%)	18 (58%)	10 (56%)	0.86	12 (52%)	16 (62%)	0.51

**Age **Mean (SD)	13.6 (± 2.5)	14.0	13.0	0.18	14.1 (± 2.7)	13.2(± 2.3)	0.20

**HIV < 50 copies/mL**	23 (47%)	12 (39%)	11 (61%)	0.13	9 (39%)	14 (54%)	0.30

**CD4% **(SD)	27.1 (± 10.2)	24.7 (± 10.4)	31.8 (± 8.3)	0.04	23.6 (± 10.5)	30.7 (± 8.8)	0.02

**Ethnicity **(Africa)	8 (16%)	3 (10%)	5 (28%)	0.10	2(9%)	6 (23%)	0.17

**eGFR at baseline **median ml/min per 1.73 m^2^(IQR)	143.6 (124.5-167.4	143.6 (123.7-165.4)	147.5 (124.5-169.6)	0.48*	135.0 (122.2-156.0)	152.6 (128.0-169.6)	0.14*

**Phosphatemia at baseline **Median mg/dl (IQR)	4.4 (3.8-5.0)	4.4 (3.8-5.0)	4.7 (3.8-5.2)	0.30*	4.4 (3.9-5.0)	4.5 (3.8-5.1)	0.44*

**Years on current regimen **mean **(SD)**	1.11 (1,64)	0,76 (1,30)	1,71 (1,99)	0.06	1 (1,41)	1,21 (1,84)	0.67

**ART regimen**							
TDF+FTC+LPV/r	10 (20%)	10 (32%)	-	-	10 (43%)		-
TDF+FTC+ATV/r	11 (22%)	11 (35%)	-	-	11 (48%)		-
TDF+FTC+Other PI/r	2 (4%)	2 (6%)	-	-	2 (9%)		-
ABC+3TC+LPV/r	6 (12%)	-	6 (33%)	-	-	6 (23%)	-
ABC+3TC+other PI	4 (8%)	-	4 (22%)	-	-	4 (15%)	-
3TC+ABC+EFV	7 (14%)	-	7 (39%)	-	-	7 (27%)	-
ABC+3TC+AZT	1 (2%)	-	1 (6%)	-	-	1 (4%)	-
TDF+ABC+EFV	1 (2%)	1 (3%)	-	-	-	1 (4%)	-
TDF+FTC+EFV	7 (14%)	7 (23%)	-	-	-	7 (27%)	-

*SD *Standard Deviation; *p *value calculated with ANOVA or with Mann Whitney test (*) and was referred to comparison between baseline variable of Group A versus Group B, or Group C versus Group D. *TDF *Tenofovir Disoproxil Fumarate; *FTC*= Emtricitabine; LPV/r = Lopinavir/ritonavir; *ATV/r *Atazanavir/ritonavir; *PI/r*= Ritonavir boosted Protease Inhibitors; *ABC*= Abacavir; *3TC*= Lamivudine; *EFV*= Efavirenz; *AZT*= Zidovudine; *OT *Other Treatments; *r*= ritonavir; *IQR *interquartile range

**Figure 1:**
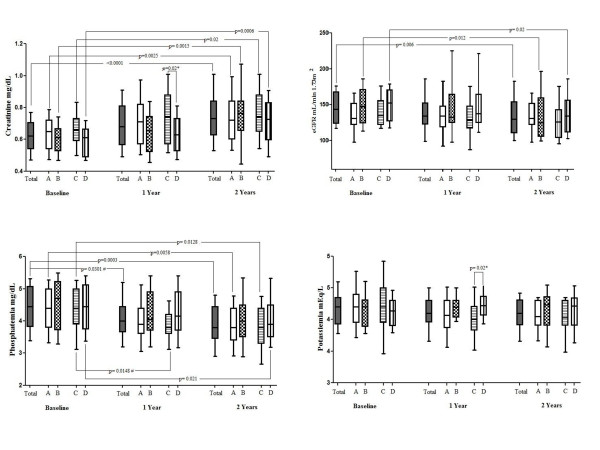
**Renal function and tubular damage markers, at baseline and after 1 and 2 years, in the entire cohort, and in each treatment group**. Patients were grouped according to the exposure to antiretrovirals: Group A = TDF, Group B = no TDF; Group C = TDF+PI, Group D = no TDF+PI. Statistical analysis related to baseline, 1 and 2 years in each group were performed through Friedman test. P-values related to analysis between group A versus group B and group C versus group D at each time point were obtained through Mann Whitney test (*), while those related to analysis between two time points among the same group with Wilcoxon matched-paired signed rank test (#).

As shown in Figure 1, in the entire cohort there was a significant increase of serum creatinine, from a median of 0.62 mg/dL at baseline to 0.73 mg/dL (*p *< 0.0001) after 2 years; eGFR decrease from a median of 143.6 at baseline to 135.6 at 1 year, and 128.9 ml/min per 1.73 m^2 ^at 2 years (*p *= 0.006). The value of phosphatemia decreased significantly from 4.4 at baseline to 4.0 at 1 year (*p *= 0.0301) and to 3.8 mg/dl at 2 years (*p *= 0.0003). The median of potassiemia in the entire cohort during the study period didn't vary significantly.

The creatinine increased significantly after 2 years in each of the groups analyzed, from +0.10 in Group A and C to +0.16 mg/dL in Group B. There were no significant differences between Group A and B, and Group C and D.

The eGFR decreased after 2 years in all groups, significantly in Group B (-23.8 ml/min per 1.73 m^2^, *p *= 0.012) and Group D (-19.1, *p *= 0.02). There were no significant differences between Group A and B, and Group C and D. Among the subgroups we observed that 7 patients on TDF+PI, 2 on PI and not in TDF, 1 in TDF and not PI, and 2 not taking nor TDF nor PI showed an increase in eGFR during the study period. On the other hand only 1 patient on TDF remained stable and all the others (37 patients) showed a decrease in eGFR. Three patients developed a mild reduction in glomerular filtration rate (60-89 ml/min per 1.73 m^2^), all were treated with both TDF and PI. Among these patients none received any other nephrotoxic drugs during the entire study period and none of them experienced infections or malignancy.

The phosphatemia decreased after 2 years in each group, significantly in Group A (-0.5 mg/dL, *p *= 0.0058) and Group C (-0.5, *p *= 0.0128). There were no significant differences between Group A and B, and Group C and D. Five patients developed a grade 1 or 2 DAIDS hypophosphatemia event; among these, 4 were treated with TDF+PI and one only with TDF. Phosphorus levels came back to normal without discontinuing the therapy. Among these patients two were HCV co infected, not taking any HCV treatment, and one patient experienced Burkitt Lymphoma and underwent chemotherapy.

## Discussion

Our study describes for the first time in a controlled study, the role of TDF and the concomitant use of TDF and PI in HIV-infected paediatric patients. The long term impact of ARV, specifically of drugs associated with renal damage on adults, is a major topic in infected children, since they are expected to have a longer duration of exposure. Moreover registration studies of antiretrovirals in children are few, with limited time of observation, and are often not focused on drugs interaction.

We reported a significant variation in the eGFR after 2 years in the entire cohort (*p *= 0.006) with a mean net loss of 12.1 ml/min per 1.73 m^2^, confirming what was reported by other studies on HIV infected adults [24,25]. We didn't find any significant eGFR variation in patients taking TDF+PI or TDF, even when compared to the group that was not treated with nor TDF nor PI, or TDF respectively. However, all three of the cases who developed a mild reduction in eGFR were on a regimen containing both TDF and PI.

This is consistent with two other studies on ARV-experienced children treated with TDF-containing regimens which didn't find evidence of impaired filtration rate, even if their sample sizes were very small (27 and 26 children respectively) and were not controlled [13,14], and with other pediatric cohort studies reporting some cases of renal toxicity associated with TDF [26-28]. Furthermore, as reported in other studies, estimated eGFR cannot be considered a sensitive indicator of early renal damage related to TDF [29], and our observation could have been affected by limited sample size. In fact, other studies on adults, with a large cohort and a higher power, found that patients treated with TDF and PI based regimens had a significantly greater decline in renal function than those taking TDF with Non-NRTIs (NNRTIs) or non-TDF-based regimens [25,30]. The role of co-administration of TDF and PI on renal function was also recently demonstrated in a large randomized clinical trial on 1858 ART-naïve adults, the ACTG 5202 study, comparing four different regimens (TDF+NNRTI; TDF+PI; other NRTIs+NNRTI; otherNRTIs+PI). In this study the only regimen with a significant reduction in estimated creatinine clearance was in the group on both TDF and PI [31].

The detection of early or mild TDF-related nephrotoxicity could be evaluated with other more specific tests for proximal tubule injury, such as serum phosphate and potassium levels.

In our study we didn't find any significant variation in potassium in all therapy groups, but there was a trend in reduction in Group C (TDF+PI) after 2 years, and this variation resulted significantly different if compared to the Group D (no TDF+PI). This seems to confirm what has been already demonstrated by observations in adults and in pediatric population on TDF [12,26].

In healthy population serum phosphorus is slightly higher in the prepubertal age, decreasing to adult values after puberty, as also considered by the age-specific DAIDS event scale. A recent case-control study in children confirmed an association between the use of TDF and hypophosphatemia, but it didn't analyze the role of concomitant use of PI [17]. In our analysis the levels of phosphatemia changed earlier (after 1 year) and significantly in the entire cohort. Moreover phosphate reduction resulted significant and worse in the group treated with TDF, and in the group treated with TDF and PI; notably four of the five patients who developed clinical hypophosphataemia were on this regimen.

The observation of a tubular damage associated with concomitant use of TDF and PI is consistent with pathogenetic cellular studies and PK analysis, showing that PI inhibition of MRP2 and MRP4 results in an increase of tubular concentration of TDF [22,23,32], and TDF clearance is slower in subjects receiving PI, with an AUC increased of 22-37%, and possible increase of mitochondrial damage [21].

However our analysis has some limitations: it is an observational study, and although the groups analyzed resulted not different for age, gender, HIV or ethnicity, other variables like immune status and disease progression, could have had a role. Furthermore we assessed renal impairment without analyzing urine tests and other more sensitive indicators such as β-2 microglobulinuria that may have shown some milder renal abnormalities. Moreover, we didn't assess the association of TDF and PI with lower bone mineral density as reported by other studies [13,17,33], and the clinical significance of kidney abnormalities observed remains not fully known. Further studies with more sensitive urine markers, bone density assays, and longer follow-up are warranted.

## Conclusion

In conclusion, this study highlights that in HIV infected children and adolescents on ART, renal function and phosphatemia decreased over time. Cases of hypophosphatemia and mild reduction of eGFR observed in patients on TDF+PI suggest an association between this ART regimen and renal function, however this appears weak and not confirmed in the subgroup analysis. New studies, with more sensitive methods and larger number, focused on the effect of these regimens on renal function of HIV infected children are warranted.

## Competing interests

The authors declare that they have no competing interests.

## Authors' contributions

GP and SB designed the study; GP and NC wrote the paper and equally contributed to this work; DA, PZ, HKT, PP provided input to the data; GP and DA performed the statistical analysis. All authors contributed to the interpretation of the data, commented the manuscript, and approved the final version.

## Pre-publication history

The pre-publication history for this paper can be accessed here:

http://www.biomedcentral.com/1471-2334/12/18/prepub
